# A Stability‐Indicating RP‐HPLC Method for Simultaneous Determination of Triclabendazole and Levamisole in Oral Suspension Formulation

**DOI:** 10.1155/ianc/3480511

**Published:** 2026-05-24

**Authors:** Abdalla Ahmed Elbashir, Khalid Malik M. Hamid

**Affiliations:** ^1^ Department of Chemistry, College of Science, King Faisal University, P. O. Box 400, Al-Ahsa, 31982, Saudi Arabia, kfu.edu.sa; ^2^ Department of Chemistry, Faculty of Science, University of Khartoum, Khartoum, Sudan, uofk.edu

**Keywords:** RP-HPLC, stability-indicating method, triclabendazole and levamisole

## Abstract

A fast, simple, and precise stability‐indicating high‐performance liquid chromatographic (HPLC) method was developed for analysis of triclabendazole (TBZ) and levamisole (LEV) in oral suspension. Using a mobile phase composed of acetonitrile, methanol, and water (50:40:10, v/v/v), with the pH adjusted to 4.6 with 0.1 M phosphoric acid. The mobile phase was filtered, degassed, and pumped at a flow rate of 1.0 mL/min. Detection was performed at 245 nm. The proposed method was validated with respect to specificity, linearity, limit of detection (LOD) and limit of quantification (LOQ), interday and intraday precision, robustness, and accuracy. The retention times for TBZ and LEV were found to be about 3.116 and 1.385 min, respectively. Calibration plots were linear with correlation coefficient of 0.9999 in the concentration range of 200–800 and 150–600 μg/mL for TBZ and LEV, respectively. The method was verified to be stability indicating by separating the active ingredients from their stressed testing degradation products. The procedure proved acceptable robustness to variations in flow rate, wavelength, and column temperature. The method was conveniently used for the analysis of TBZ and LEV in oral suspension.

## 1. Introduction

Benzimidazoles are heterocyclic compounds consisting of a benzene ring fused with an imidazole ring, which are widely used as pesticides and veterinary drugs [1]. Recently, benzimidazoles are commonly utilized as veterinary drugs, for example, triclabendazole (TBZ), albendazole, levamisole (LEV), fenbendazole, flubendazole, mebendazole, and oxibendazole [2, 3].

**FIGURE 1:**
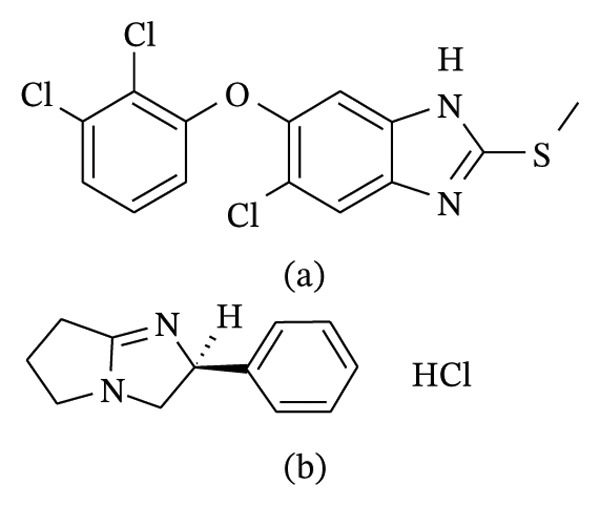
Chemical structures of (a) levamisole hydrochloride and (b) triclabendazole.

TBZ is a white crystalline solid, chemically described as 6‐chloro‐5‐(2,3‐dichlorophenoxy)‐2‐methylsulfanyl‐1H‐benzimidazole (Figure 1(a)). TBZ was the first marketed benzimidazole over 50 years ago [4, 5]. TBZ is an anthelmintic utilized for control of liver fluke, *Fasciola gigantica*, and *F. hepatica* in cattle, goat, and sheep [6, 7]. TBZ is formulated in an oral suspension for cattle, sheep, and, in some countries, goats as well as in pour‐on formulations for cattle. TBZ is also used for the treatment of fascioliasis in humans [8, 9].

LEV is a white or almost white, crystalline powder, chemically known as (6S)‐6‐phenyl‐2, 3, 5, 6‐tetrahydroimidazo thiazole hydrochloride (Figure 1(b)) [1, 3]. It is freely soluble in water and ethanol and slightly soluble in methylene chloride. LEV was discovered in 1966 and is included in the list of essential medicines of the WHO, as safe and effective medicines needed in a health system [10].

Combined chemotherapy especially with drugs of different chemical classes is one of the important strategies for preventing development of resistant parasites [11–14]. Recently, Tabari et al. have demonstrated that the combination of TBZ + LEV resulted in higher efficacy and earlier improvement of liver conditions in sheep naturally infected with *Fasciola* sp. [15].

The official method for the determination of TBZ and LEV in individual formulation is by titration [16–18]; however, there is no official protocol for simultaneous analysis of TBZ and LEV. Several procedures were reported for the determination of TBZ; these methods include spectrophotometric methods [19], spectrofluorometric methods [20], and high‐performance liquid chromatographic (HPLC) methods with UV detection [21, 22] and mass spectrometric detection [23, 24]. Commercial formulations containing combination of TBZ and LEV for treatment of liver flukes and gastrointestinal nematodes in cattle and sheep are available in the market [25, 26]. Therefore, analysis of drug combination is of great importance for commercial production. Spectrophotometric methods for simultaneous determination of TBZ and LEV in veterinary tablets were reported [25, 26].

The determination of LEV in various formulations in combination with other active ingredients and their metabolites has been accomplished by HPLC [27–31]. To the best of our knowledge, the simultaneous determination of the binary mixture of TBZ and LEV by HPLC was not investigated in the literature. In this study, a simple, selective, precise, and accurate stability‐indicating HPLC method for analysis of TBZ and LEV in oral suspension was developed and validated.

## 2. Materials and Methods

### 2.1. Instrumentation

The analysis was performed using a Shimadzu HPLC system (Japan) equipped with a Prominence autosampler (SIL‐20AC), UFLC pump (LC‐20AB), detector (SPD‐20AV), degassing unit (DGU‐20A3R), and column oven (CTO‐20A). Data acquisition was carried out with Shimadzu LC‐Solution software.

### 2.2. Materials and Chromatographic Conditions

Methanol and acetonitrile (HPLC grade) were obtained from Scharlau‐chemical (Spain). Phosphoric acid (85% w/w) was obtained from Sigma‐Aldrich (St. Louis, USA). Oral suspension claimed to contain LEV 37.5 mg/L and TBZ 50 mg/L, as active ingredients, was purchased from the local market. Purified water was obtained from Bash Pharma Co. Ltd (Sudan). 0.45‐μm nylon filters were obtained from Vivid Separation and Filtration, Jordan. The chromatographic separation was performed on a stainless‐steel C8 column (150 mm × 4.6 mm, 5.0 μm particle size) using an injection volume of 10 μL. The mobile phase consisted of acetonitrile:methanol:purified water (50:40:10, v/v/v) adjusted to pH 4.6. The analysis was conducted at a flow rate of 1.0 mL/min, with detection at 245 nm..

### 2.3. Preparation of Standard Stock Solutions and Oral Suspension Solution

50 mg of TBZ and 37.5 mg of LEV working standard were accurately weighed into 100 mL volumetric flask, dissolved in acetonitrile, and completed to the mark with same solvent and mixed. Series of standard solutions were prepared by appropriate dilution. The commercial oral suspension was thoroughly homogenized by vigorous shaking prior to sampling. An accurately measured 1.0‐mL aliquot was transferred into a 100‐mL volumetric flask, diluted with acetonitrile, and sonicated for an appropriate time to ensure complete dispersion and extraction of the analytes. The solution was then brought to volume with the same solvent, mixed well, and filtered through a 0.45‐μm nylon membrane filter prior to injection into the HPLC system.

### 2.4. Stress Testing

Stress testing of pharmaceutical formulation is utilized to identify degradation products, furnish an evidence of the stability active ingredient, and validate the stability and specificity of the analytical method [32]. To study stress testing, a solution of the pharmaceutical formulation containing TBZ and LEV at a concentration of 50 mg/mL and 37.5 mg/mL, respectively, was used. The solutions were subjected to stress conditions using 0.1 M hydrochloric acid and 0.1 M sodium hydroxide for 5 min, 3% hydrogen peroxide for 30 min, heating at 60°C for 60 min, and exposure to UV light for 180 min. The stressed samples were analyzed, and the chromatograms of the stress samples, reference sample, and placebo were compared.

## 3. Results and Discussion

### 3.1. Optimization of Chromatographic Conditions

**FIGURE 2:**
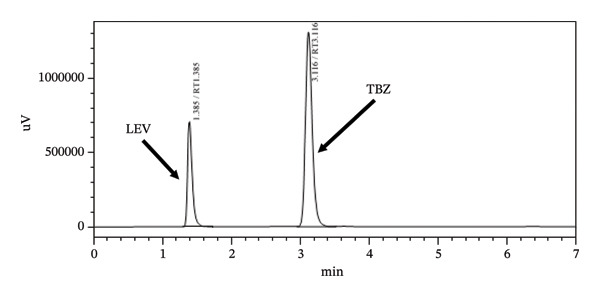
Chromatogram of standard solution of TBZ and LEV at optimized HPLC conditions. The concentration of TBZ and LEV is 800 and 600 μg/mL, respectively.

Several experiments were conducted using different proportions of organic solvents and varying flow rates to achieve optimal resolution, shorter retention times, and symmetrical peak shapes for the analytes. At the start, mobile phase consisting of acetonitrile:methanol:purified water (60:35:5) (V:V:V) at pH 4.6 was employed; then, the % of acetonitrile was decreased and methanol was increased to 55:40:5 (V:V:V). Finally, 50:40:10 (V:V:V) at pH 4.6 was used. Within all tried experiments, mobile phase consisting of acetonitrile:methanol:purified water (50:40:10) (V:V:V) at pH 4.6 was found to be appropriate since good resolution with fast separation was obtained. Typical chromatogram for the mixture of the two drug standards obtained at the optimized conditions is shown in Figure 2. TBZ is a relatively nonpolar benzimidazole derivative, whereas LEV is more polar due to its secondary amine and amide functionalities. On the reversed‐phase C8 column, the retention is primarily governed by hydrophobic interactions. TBZ, being more nonpolar, exhibits stronger interactions with the hydrophobic C8 stationary phase, resulting in longer retention. In contrast, the more polar LEV interacts less with the stationary phase and elutes earlier. These differences in polarity and hydrophobicity explain the observed separation and selectivity under the optimized mobile phase conditions.

### 3.2. System Suitability

System suitability investigations are essential in HPLC method development, and they were employed to confirm that the presented procedure was capable of producing good resolution between the peaks with high reproducibility. The system suitability and optimization of the chromatographic conditions were evaluated through replicate injections (*n* = 5) of freshly prepared standard solutions. Key chromatographic parameters, including peak area, resolution (Rs), tailing factor, and theoretical plate number, were assessed for each analyte. In addition, different solvent compositions were systematically investigated during method development, and the corresponding chromatographic performance such as resolution, selectivity, and peak symmetry was carefully monitored. A standard mixture containing both TBZ and LEV was analyzed under the optimized conditions, resulting in complete and well‐resolved peaks for both compounds. The obtained chromatographic parameters, which support the selection of the optimized mobile phase, are summarized in Table 1. The theoretical plate number (N) was calculated according to the United States Pharmacopeia (USP) method using retention time and peak width parameters obtained from the chromatographic data via the instrument software, confirming compliance with established chromatographic system suitability criteria.

**TABLE 1:** System suitability results of the presented method.

	**Tailing factor**	**Resolution**	**Theoretical plates**	**RSD peak area**

TBZ	0.95	5.8	47,392	0.63
LEV	0.97	5.8	39,268	0.48
Required limits	< 1.5	Rs > 2	> 2000	RSD < 2

### 3.3. Method Validation

Method validation is the way of verifying that the procedure is adequate for its planned goal. For pharmaceutical methods, recommendations from the International Conference on Harmonization (ICH) and USP give a framework for executing such validations.

#### 3.3.1. Specificity

**FIGURE 3:**
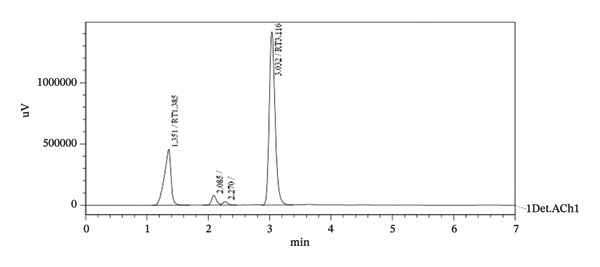
Chromatogram of oral suspension sample solution of TBZ and LEV at optimized HPLC conditions.

The capability of a method to differentiate between the analyte of interest and other constituents that are existing in the sample is known as specificity. Placebo of the oral suspension equivalent to the sample volume was taken, and the solution was prepared as for the sample solution. This solution and sample solution were analyzed employing the proposed procedure. No interference from placebo solution was seen at the retention time of the two active ingredients. This is enough to confirm the specificity for excipients, as shown in Figure 3.

#### 3.3.2. Linearity, Limit of Detection (LOD), and Limit of Quantification (LOQ)

According to ICH guidelines, at least five concentrations of standard must be studied and a graph of the detector response versus the concentration must be established [32, 33]. To investigate the linearity range of TBZ and LEV, serial dilutions were made to give working standard solution in the concentration range of 200–800 μg/mL and 150–600 μg/mL for TBZ and LEV, respectively. The statistical linear regression data measured are presented in Table 1. The procedure was found to be linear in the concentration range examined for the two active ingredients. Calibration curves obtained are shown in Figure 4.

FIGURE 4Calibration curves of (a) TBZ and (b) LEV.

**Figure:**
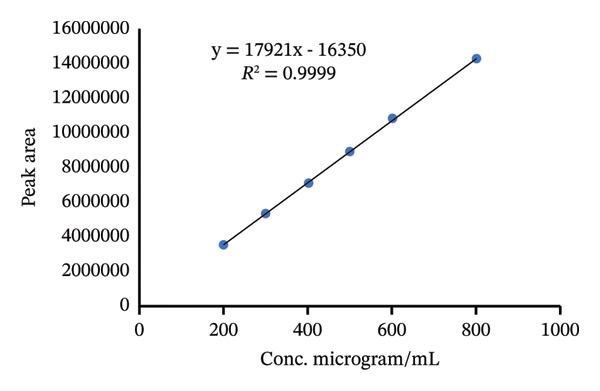
(a)

**Figure:**
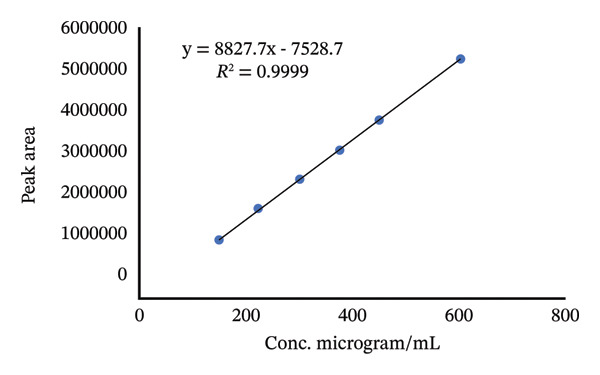
(b)

The LODs and LOQs were evaluated according to 3.3 *σ*/s and 10 *σ*/s formulas, respectively [34, 35], where *σ* and s are the standard deviation of the intercept and the slope, respectively. The LODs and LOQs were measured to be 9.28, 6.7 μg/mL and 28.12, 20.4 μg/mL for TBZ and LEV, respectively (Table 2).

**TABLE 2:** Parameters of linearity data for TBZ and LEV.

Parameter	TBZ	LEV
Linear range (μg/mL)	200–800	150–600
Intercept	16350	7528.7
Slope	17921	8827.7
Correlation coefficient	0.9999	0.9999
LOD (μg/mL)	9.28	6.7
LOQ (μg/mL)	28.12	20.4

#### 3.3.3. Precision

The intraday precision and interday precision were used to measure the precision of the developed method. The intraday precision was measured by analyzing six replicates on the same day, whereas the interday precision was carried out over six consecutive days [30, 31]. The data achieved are presented in Table 3. The relative standard deviation (RSD) for intraday and interday was calculated to be less than 2% revealing that the procedure was precise.

**TABLE 3:** Intraday test (repeatability) and interday precision studies for the determination of TBZ and LEV by the proposed method.

Intraday precision (*n* = 6)	Assay % of TBZ	RSD%	Assay % of LEV	RSD%
	105.62	0.44	103.10	0.41
Interday precision (*n* = 36)	105.56	1.19	104.74	1.56

#### 3.3.4. Accuracy

The accuracy was examined by spiking the pharmaceutical formulation with working standards of TBZ and LEV. Standards were added to samples at three concentration levels, 40%, 100%, and 160%, and injected in triplicate. The % recovery was calculated and considered as the accuracy (Table 4). The recoveries of the two active ingredients were within the stated ICH guidelines [36]. The high percentage of the recoveries obtained indicates the accuracy of the proposed method.

**TABLE 4:** Recovery studies for the determination of TBZ and LEV by the proposed method.

Analyte	Sample content (μg/mL)	Average amount found (μg/mL)	Recovery %	RSD%
TBZ	40	40.4	101.1	1.41
	100	100.8	100.8	0.72
	160	158.8	99.3	0.32

LEV	40	40	99.9	1.48
	100	101	101	0.77
	160	158.6	99.2	0.63

#### 3.3.5. Robustness

Robustness tests were carried out to examine the reliability of results when small changes in experimental parameters such as flow rate, wavelength, and column oven temperature were slightly changed. Only one parameter was altered at a time. No pronounced variation was observed between the results, demonstrating the robustness of the method (Table 5).

**TABLE 5:** Influence of small variation in the assay condition on the analytical performance of the proposed HPLC method for the determination of TBZ and LEV.

Analyte	HPLC parameters	Retention time	Theoretical plate	Tailing factor
TBZ	Optimum	3.116	4472	1.295
	Wavelength: 247 Wavelength: 243	3.059	4427.49	1.303
		3.084	4127.34	1.313
	Flow rate: 1.2 mL Flow rate: 0.8 mL	2.548	3885.51	1.299
		3.21	4368	1.362
	Column oven: 30°C Column oven: 25°C	3.079	4459.62	1.308
		3.33	4145	1.341

LEV	Optimum	1.385	1658	1.665
	Wavelength: 247 Wavelength: 243	1.386	1752.042	1.669
		1.301	1715	1.565
	Flow rate: 1.2 mL Flow rate: 0.8 mL	1.161	1530.27	1.673
		1.395	1662.2	1.536
	Column oven: 30°C Column oven: 25°C	1.298	1764.19	1.692
		1.391	1684.24	1.614

### 3.4. Stress Testing

Forced degradation examinations were carried out to prove the validity of the presented procedure in terms of specificity. Degradation tests were carried out employing the following conditions, base, acid, oxidation, UV‐light, and heat. HPLC chromatograms of pharmaceutical formulation degradation studies are shown in Figures 5(a), 5(b), 5(c), 5(d), 5(e), and 5(f). It is clear from the chromatograms that there is no interference between the two active ingredients and degradation products. Peak purity was evaluated using a photodiode array (PDA) detector by assessing spectral homogeneity across the analyte peaks under all stress conditions; in all cases, the purity angle was found to be less than the purity threshold, confirming the absence of coeluting degradation products and supporting the stability‐indicating nature of the method.

FIGURE 5Chromatograms at different degradation and stability conditions. (a) Placebo sample. (b) TBZ + LEV sample with 0.1 M hydrochloric acid for 5 min. (c) TBZ + LEV sample with 0.1 M sodium hydroxide for 5 min. (d) TBZ + LEV sample with hydrogen peroxide 3% for 30 min. (e) TBZ + LEV sample at 60°C, for 60 min. (f) TBZ + LEV sample at UV‐light, for 180 min.

**Figure:**
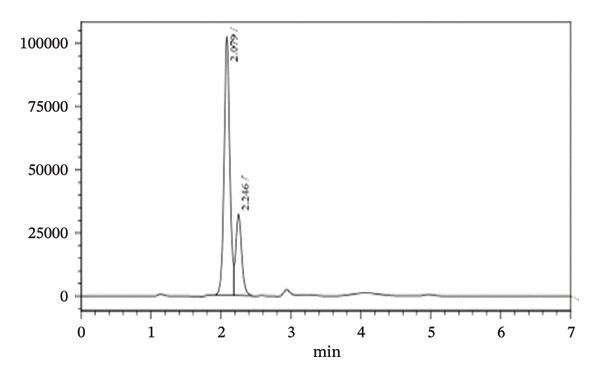
(a)

**Figure:**
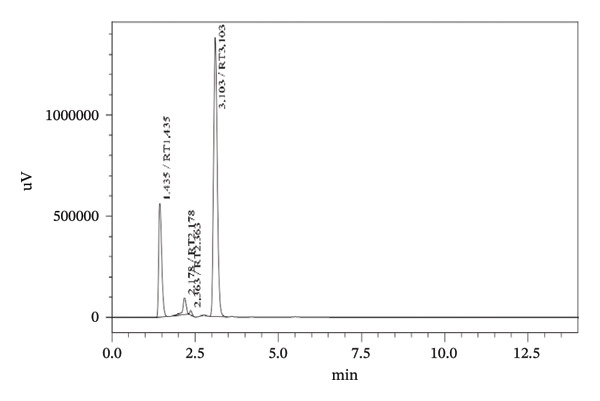
(b)

**Figure:**
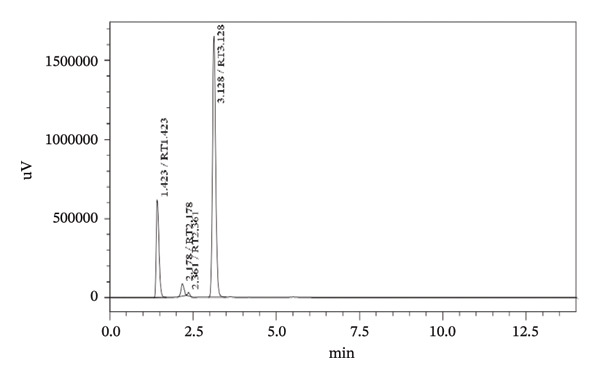
(c)

**Figure:**
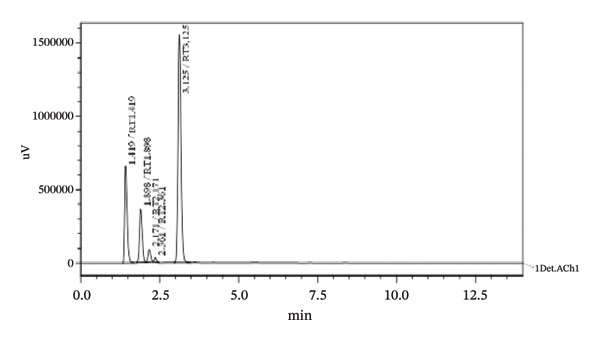
(d)

**Figure:**
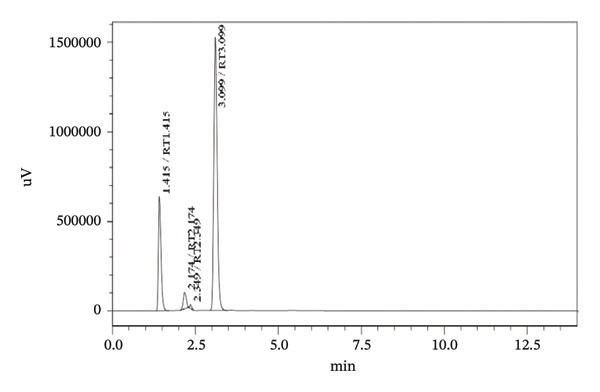
(e)

**Figure:**
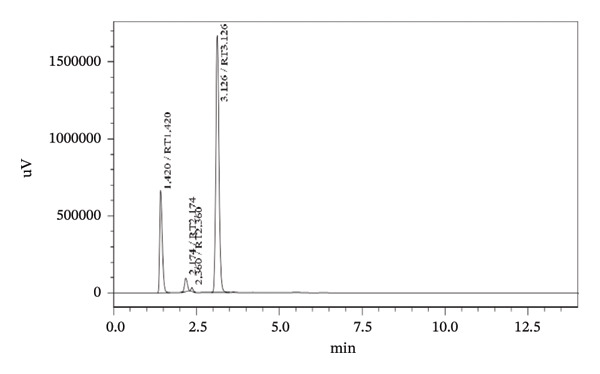
(f)

### 3.5. Analysis of the Pharmaceutical Preparation

The validated HPLC method was employed for analysis of TBZ and LEV in an oral suspension dosage form (Trisole oral suspension). TBZ and LEV were eluted at their exact retention times as presented in Figure 3. Peaks from other excipients were not seen within the retention times of TBZ and LEV. The analysis results showed acceptable precision and accuracy as shown from % recovery, SD, and RSD% values (Tables 3 and 4). The pharmacopoeia mentioned assay limits of 90%–110% for each drug constituent [35] (Table 3). The results obtained demonstrated that the presented procedure is appropriate for analysis of these two drugs with an acceptable level of selectivity, precision, and accuracy.

## 4. Conclusions

The present work demonstrated the validation of stability‐indicating HPLC procedure for the simultaneous analysis of TBZ and LEV in pharmaceutical preparation for the first time. The procedure was validated according to ICH guidelines. The method can be used for routine analysis of TBZ and LEV in pharmaceutical formulations in quality control and industry.

## Funding

This study was supported by King Faisal University, KFU260848.

## Conflicts of Interest

The authors declare no conflicts of interest.

## Data Availability

The data that support the findings of this study are available on request from the corresponding author. The data are not publicly available due to privacy or ethical restrictions.
